# Protective Effects of Polysaccharides in Neurodegenerative Diseases

**DOI:** 10.3389/fnagi.2022.917629

**Published:** 2022-07-04

**Authors:** Yinying Wang, Rongsha Chen, Zhongshan Yang, Qian Wen, Xia Cao, Ninghui Zhao, Jinyuan Yan

**Affiliations:** ^1^The Central Laboratory of the Second Affiliated Hospital, Kunming Medical University, Kunming, China; ^2^Yunnan Provincial Key Laboratory of Molecular Biology for Sino Medicine, Yunnan University of Chinese Medicine, Kunming, China; ^3^The Neurosurgery Department of the Second Affiliated Hospital, Kunming Medical University, Kunming, China

**Keywords:** mechanism, polysaccharides, Alzheimer’s disease, Parkinson’s disease, Huntington’s disease

## Abstract

Neurodegenerative diseases (NDs) are characterized by progressive degeneration and necrosis of neurons, including Alzheimer’s disease (AD), Parkinson’s disease (PD), Huntington’s disease and others. There are no existing therapies that correct the progression of these diseases, and current therapies provide merely symptomatic relief. The use of polysaccharides has received significant attention due to extensive biological activities and application prospects. Previous studies suggest that the polysaccharides as a candidate participate in neuronal protection and protect against NDs. In this review, we demonstrate that various polysaccharides mediate NDs, and share several common mechanisms characterized by autophagy, apoptosis, neuroinflammation, oxidative stress, mitochondrial dysfunction in PD and AD. Furthermore, this review reveals potential role of polysaccharides *in vitro* and *in vivo* models of NDs, and highlights the contributions of polysaccharides and prospects of their mechanism studies for the treatment of NDs. Finally, we suggest some remaining questions for the field and areas for new development.

## Introduction

Neurodegenerative diseases (NDs) are a series of the central nervous system (CNS) disorders that cause a slowly progressive loss of function of specific neuron populations and their connections including sporadic and hereditary forms (Reith, 2018). Most evidences suggest that approximately 25% of Alzheimer’s disease (AD) is familial and 75% is non-familial (Bird, 2018). Approximately 5–10% of Parkinson’s disease (PD) is inherited in an autosomal dominant, autosomal recessive, or even X-linked pattern of inheritance (Lesage and Brice, 2009). Moreover, the incidence of genetically confirmed Huntington’s disease (HD) in symptomatic individuals with no known family history of HD may be as high as 8% of all individuals with HD (Margolis and Ross, 2003). A number of NDs have an underlying genetic cause, with a 50% recurrence risk when inheritance is autosomal dominant (Roberts et al., 2020). The major pathological hallmarks of NDs are the accumulation and aggregation of proteins in specific neurons aggregation in the CNS. The prevalence of NDs is expected to rise with the increasing life expectancy. The complex and diverse pathological features, unclear molecular mechanisms, limited clinical examination options, difficulties in early diagnosis, and lack of specificity in treatment have introduced substantial social and economic burdens (Chen-Plotkin, 2014; Kwon et al., 2016; Marsh, 2019; Muddapu et al., 2020). There are various theories on the pathogenesis of AD, the dominant theories include the amyloid beta (Aβ) toxicity hypothesis, the microtubule-associated protein – tau protein functional abnormality hypothesis (Morris et al., 2014), the vascular gene hypothesis (De La Torre, 2010), and the gene mutation hypothesis. AD patients are characterized by accumulation of Aβ into senile plaques and hyperphosphorylated tau into neurofibrillary tangles (Spires-Jones and Hyman, 2014). AD impairs memory and cognitive judgment and is often accompanied by mood swings, disorientation and eventually delirium (Cass, 2017). Despite increases in medication dosage, the efficacy of pharmacological treatments still reduces with disease progresses. The current drugs for AD treatment include neurotransmitter agents such as cholinergic inhibitors (including tacrine, donepezil, rivastigmine, and galantamine; Sharma, 2019), drugs directed at β1 amyloid (E2069, MK-8931; Blume et al., 2018), antioxidant drugs (including monoamine oxidase inhibitors and melatonin; Cardinali et al., 2014; Ostadkarampour and Putnins, 2021), and calcium channel blockers (including nilvadipine, nimodipine, and flunarizine; Nimmrich and Eckert, 2013). Moreover, PD is a severe neurodegenerative disorder that affects around 2–3% of the population over 65 years old (Poewe et al., 2017). PD is characterized by the loss of dopaminergic (DA) neurons in the pars compacta of the substantia nigra and by accumulation of misfolded α-synuclein (α-syn; Balestrino and Schapira, 2020); its cardinal motor symptoms are bradykinesia, rigidity, postural instability, and tremor (Dickson, 2018). Despite significant progress made over the past several decades, PD is still an incurable disorder. The currently available therapeutic approaches focus on stimulation of DA signaling, such as levodopa (L-DOPA), DOPA decarboxylase inhibitors (Bandopadhyay et al., 2022), catechol-O-methyltransferase inhibitors (Salamon et al., 2022), dopamine agonists (Jenner, 2002), and inhibitors of the enzyme monoamine oxidase type B (Nagatsu and Sawada, 2006). Of these, L-DOPA remains the single most effective therapeutic agent for PD patients (Ngwuluka et al., 2010). HD is characterized by a general shrinkage of the brain and degeneration of the striatum (caudate nucleus and putamen) due to the mutation of *Huntingtin* (*HTT*) gene (Jimenez-Sanchez et al., 2017); The symptoms of HD encompasses psychiatric conditions, cognitive defects, motor impairment (e.g., chorea; Huang et al., 2016). Tetrabenazine and Deutetrabenazine, both approved by the Food and Drug Administration (FDA; Potkin and Potkin, 2018), are inhibitors of the vesicular monoamine transporter type 2, and functions through depletion of dopamine in the presynaptic terminals to improve chorea (Dean and Sung, 2018; Kumar et al., 2020), The different drug therapies available for the treatment of NDs are shown in (Table 1).

**TABLE 1:** The different drug therapies available for the treatment of NDs.

NDs	Medicine	Treatment mechanisms and disadvantages	References
AD	Cholinergic inhibitors (tacrine, donepezil, rivastigmine, and galantamine)	Increase the cholinergic levels in the brain by inhibiting the biological activity of AChE, and may cause adverse side effects (liver damage, nausea, vomiting, diarrhea, neuromuscular transmission and respiratory paralysis)	Sharma, 2019
	β1 amyloid (E2069, MK-8931)	Lower cerebral Aβ concentrations, may cause adverse side effects (hypomyelination, seizures, axon guidance defects, memory deficits, neurogenesis abnormalities)	Blume et al., 2018
	Antioxidant drugs (Monoamine oxidase inhibitors and melatonin)	Anti-inflammatory effects, cause adverse side effects (hepatotoxicity and hypertensive crisis, neuromuscular, autonomic, and mental status symptoms), and melatonin exerts its inhibitory effect on the generation of Aβ remains undefined	Cardinali et al., 2014; Ostadkarampour and Putnins, 2021
	Calcium channel blockers (nilvadipine, nimodipine, and flunarizine)	Decrease calcium influx through the plasma membrane and impairment of synapse physiology, protect AD cells from Aβ oligomer production	Nimmrich and Eckert, 2013
PD	Levodopa (L-DOPA), DOPA decarboxylase inhibitors	L-DOPA treatment is highly efficient, and is converted to DA and stored in the vesicles of presynaptic DA neurons, prolong use gives rise to motor abnormalities, including dyskinesia	Bandopadhyay et al., 2022
	Catechol-O-methyltransferase inhibitors	Inhibit of catechol-O-methyl transferase enzyme, and results in a higher levodopa concentration in the blood without peripheral degradation to 3-O-methyldopa, increase the bioavailability of L-DOPA, and may cause adverse side effects (liver toxicity, nausea, drowsiness, insomnia, dizziness, hallucination)	Salamon et al., 2022
	Dopamine agonists	Act on either D1-like or D2-like dopamine receptors, and the multiple receptor subtypes present in the brain, produce dyskinesia identical to that of L-dopa	Jenner, 2002
	Inhibitors of the enzyme monoamine oxidase type B	Degrade the neurotransmitter DA that is deficient in the nigro-striatal region in PD, and forms H_2_O_2_ and toxic aldehyde metabolites of DA, inhibit of DA degradation	Nagatsu and Sawada, 2006
HD	Inhibitors of the vesicular monoamine transporter type 2 (Tetrabenazine and Deutetrabenazine)	Inhibit vesicular monoamine transporter (VMAT) type 2 and consequently decrease available dopamine in the synapse and interaction with postsynaptic dopamine receptors	Potkin and Potkin, 2018

The currently available treatments for NDs are primarily focused on symptom management and no cure is yet available for this devastating disorder, the search for new and effective NDs therapies remains a priority. Herein, this review provides a summary on the pathogenesis and treatment of NDs, and summarizes the application and limitations of different forms of polysaccharides in the most common NDs.

## Polysaccharides

Polysaccharides are naturally and synthetically active macromolecular substances formed by more than ten monosaccharides connected by glycosidic bonds. Natural polysaccharides are widely present in plants, animals, algae, and microorganisms. The polysaccharides are regarded as potential useful agent for the prevention of neuronal damage, and have received considerable attention for wide-ranging bioactivity (Dhahri et al., 2022). Polysaccharides have other beneficial properties including biodegradability, high stability and low toxicity, solubility in water, higher degrees of swelling capability, etc. (Mozammil Hasnain et al., 2019). Moreover, polysaccharide nanoparticles improve the bioavailability and bioactivity of functional ingredients, such as *Ganoderma lucidum*, *Momordica charantia* polysaccharides (Qin et al., 2018) and chitosan (Xue et al., 2020). The polysaccharides mainly include cellulose, chitin and other polysaccharides, and are classified into two major types: indigestible and digestible. The digestible polysaccharides are hydrolyzed to sugar subunits in the stomach, absorbed by the small intestine. Among indigestible polysaccharides, non-fermentable polysaccharides are unable to hydrolyze in the stomach and the small intestine, go through the large intestine, and are finally excreted out in the forms of waste or feces. Only the fermentable and indigestible polysaccharides are metabolized to produce various metabolites by the host intestinal microbiota, such as short chain fatty acids (SCFAs); then these metabolites modulate the host metabolism and pathophysiology process (Ahmadi et al., 2017). The polysaccharides have been widely concerned due to their immunomodulatory function. The polysaccharides bind to polysaccharides receptors, including Toll-like receptor family (TLR), C-type lectin receptor family, complement receptors (CR3), scavenger receptor (SR), mannose receptor family (MR) and so on; these receptors are bound to activate intracellular signaling pathways that generate immune response, such as effects on macrophages, T-lymphocytes, B-lymphocytes, dendritic cells, red blood cells and natural killer cells, affect complement system, and regulate cytokines secretion (Jiang et al., 2010; Zhao et al., 2020).

Recently, polysaccharides have become more prominent as energy sources and supporting tissue structures. The polysaccharides possess many several physiological functions and biological activities, including immune regulation (Lin et al., 2021), anti-tumor (Sha et al., 2022), anti-radiation protection (Li X. et al., 2021), anti-diabetic (Wang P.-C. et al., 2016), anti-virus (Zhang et al., 2022), anti-bacteria (Wang et al., 2021), anti-fatigue functions (Shen et al., 2021), anti-aging (Zhu et al., 2020), and regulation of ubiquitination (Yan et al., 2022), iron metabolism (Ren et al., 2021), intestinal flora (Cai et al., 2021), estrogen (Hwang et al., 2020), and autophagy (Wu et al., 2021b) and so on. Polysaccharides also reduce Aβ deposition (Li G. et al., 2021), inhibit neurotoxicity (Huang et al., 2021), reduce oxidative stress (Liu et al., 2020), exert anti-inflammatory actions (Wen et al., 2022), and inhibit neuronal apoptosis (Guo et al., 2016). These functions and mechanisms of polysaccharides suggest that it is possible for prevention and treatment targets in NDs. In addition, polysaccharides also have some negative effects, such as, lipopolysaccharide (LPS) and zymosan. Zymosan represents an acute inflammation model widely used for the quantification of neutrophils and inflammation-related soluble factors (Cash et al., 2009). LPS is an endotoxin in the outer membrane of most gram-negative bacteria, and is a direct glial-mediated inflammatory stimulus (Kim et al., 2019). LPS is used to model neuroinflammation associated with NDs *via* targeting TLR4 that is primarily expressed on microglial in CNS to produce proinflammatory cytokines (Page et al., 2022). LPS also stimulates glial cells, leads to neuroinflammation for modeling inflammation-mediated DA neurodegeneration in PD animals (Dutta et al., 2008). Nevertheless, low-dose LPS treatment can awake the peripheral immune system, finally delay the further progression of HD (Lee et al., 2018).

In dextran sodium sulfate-induced ulcerative colitis mice, *Ficus carica* polysaccharide changed the abundance of gut microbiota, suppressed the infiltration of inflammatory cell and cytokine formation to prevent the disease development (Zou et al., 2020). Another study demonstrated that *Lycium barbarum* polysaccharide reduced myocardial damage by regulating the intestinal microbiome and fecal metabolome (Zhang et al., 2020b). *Bacteroides fragilis* is an important member of the human gut microbiome, which regulated specific *capsular* polysaccharides to attract IgA binding and deploy specific capsules for immune attraction, potentially enabling stable mucosal colonization and clear pathogens (Donaldson et al., 2018). In nervous system, these receptors, including TLR2, TLR3, TLR4, MR, and SR, are also widely expressed and participate in CNS injury and NDs (Burudi and Régnier-Vigouroux, 2001; Husemann and Silverstein, 2001; Marsh and Stenzel-Poore, 2008; Perez-Pardo et al., 2019; Zhou et al., 2019). If the polysaccharides cross the blood-brain barrier (BBB), they as extrinsic ligands bind to the above receptors to mediate the intracellular signaling in NDs. Due to the poor BBB permeability of most polysaccharides, they may regulate intestinal flora and related metabolic products to exert neuroprotective effects.

**FIGURE 1:**
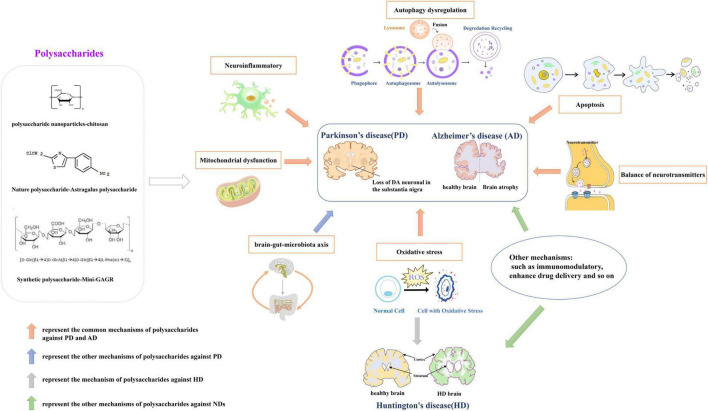
The neuroprotective mechanisms of polysaccharides involved in neurodegenerative diseases (NDs). Drawn by Chen Shen, The Central laboratory of the Second Affiliated Hospital, Kunming Medical University.

In a mouse PD model, *Astragalus* polysaccharides decreased the bax/bcl2 ratio, reversed mitochondrial structural damage and attenuated motor dysfunction (Liu H. et al., 2018). *G. lucidum* polysaccharides regulated expression of apoptosis-associated proteins, inhibited oxidative stress-induced neuronal apoptosis, and had significant neuroprotective effects in cerebellar granule cells (Sun et al., 2017). Wang et al. reported that sulfated hetero-polysaccharides extracted from *Saccharina japonica* may had a “therapeutic anti-apoptosis” utility for PD by enhancing the phosphorylation of PI3K/Akt signaling pathway and impairing caspase-3 levels in H_2_O_2_-induced SH-SY5Y cells (Wang et al., 2017). *Angelica* polysaccharide significantly alleviated LPS-induced PC-12 cell viability inhibition, apoptosis and expression of inflammatory cytokines, inactivated NF-κB pathway by down-regulating miR-223 (Li R. et al., 2018). *Astragalus* polysaccharide activated the PI3K/AKT/mTOR to increase autophagy, finally elevated cell viability in PD cell model (Tan et al., 2020). Currently, it has reported that the common mechanisms of polysaccharides against PD and AD involve in neuroinflammation, oxidative stress, autophagy, apoptosis, mitochondrial dysfunction. And, the mechanisms of HD primarily involved in oxidative stress and neurotoxicity. Figure 1 shows the neuroprotective mechanisms of polysaccharides involved in NDs.

## The Role of Polysaccharides in Neurodegenerative Diseases

### Parkinson’s Disease

Parkinson’s disease is the second most frequent neurodegenerative disorder of aging, affecting 7–10 million people worldwide, is diagnosed clinically based on typical motor symptoms, including bradykinesia, rigidity, abnormal posture, and resting tremor. Exposure to pesticides or herbicides, carbon monoxide, organic solvents, carbon disulfide, well water, rural environment, plant-derived toxins, and infection are all considered as causes of PD. The pathological feature of PD is the loss of DA neurons, and abnormal accumulation of α-syn. α-syn is a small (140 aa) protein generally enriched in the presynaptic compartment that regulate vesicle dynamics and trafficking, and neurotransmitter release (Emamzadeh, 2016).

Although the etiology of PD remains unclear, the death of DA neurons during PD progress is revealed to be associated with the abnormal aggregation of α-syn (Luk et al., 2012), mitochondrial dysfunction (Mandemakers et al., 2007), overactivated oxidative stress (Ren and Butterfield, 2021), apoptosis (Liu J. et al., 2018), or autophagy (Hou X. et al., 2020), microglia response caused by neuroinflammation (Tiwari and Pal, 2017), microbiota-gut-brain axis (Menozzi et al., 2021), autosomal dominant and recessive sexual heredity-related genetic changes (Filatova et al., 2014), and other factors are closely related. PD is a significant disease that affects human health and life, and brings substantial social and economic burdens to families and society. Since no viable treatment is available for PD to cease or reverse the disease progression, there is an urgent need for novel therapeutic approaches.

Oxidative stress is caused by increasing reactive oxygen species (ROS) product and weakening antioxidant capacity, damaging lipids, proteins, and DNA. ROS in the physiological state are important redox messengers that regulate various signaling pathways and play a crucial role in regulating cellular metabolism, post-transcriptional modification of proteins, and antioxidant defense mechanisms. However, excessive ROS damage lipids, proteins and DNA, and inhibit normal cell function. Oxidative stress is a state of stress damage caused by the imbalance of oxidation and anti-oxidation in the cell (Dröge, 2002). For example, in 1-methyl-4-phenyl-1,2,3,6-tetrahydropyridine (MPTP)-induced mouse PD models, *M. charantia* polysaccharides inhibited oxidative stress products in the brain, thereby increased DA levels, alleviated the impairment of coordination and motor ability by regulating the TLR4/MyD88/NF-κB pathway (Guo et al., 2021). A blue-green alga *spirulina platensis* polysaccharide attenuated the reduction in tyrosine hydroxylase and the dopamine transporter expression (DA-specific markers) *via* increasing in the activities of superoxide dismutase (SOD), and glutathione peroxidase (GSH-Px) in same mouse PD models (Zhang et al., 2015), SOD and GSH-px are important endogenous antioxidants. In 6-hydroxydopamine (6-OHDA) – induced mouse PD model, *Antrodia camphorata* polysaccharide inhibited the activation of ROS *via* increasing the activity and expression of antioxidant enzymes, ultimately reduced DA neuronal damage in the substantia nigra, improves motor performance (Han et al., 2020). Many accumulated evidences show that a prominent feature in many NDs is excessive ROS, it would attack lipid membranes, proteins and DNA (Wu et al., 2006), and eventually contribute to the injury and death of neuronal cells. In H_2_O_2_ – induced PC12 cells, wild desert plant – drived *Cynomorium songaricum* Rupr polysaccharide maintained the antioxidant system, protected the cells against oxidative damage (Wang F. et al., 2016). A selenium polysaccharide from *Platycodon grandiflorum* attenuated intracellular ROS formation to inhibit the decrease of cell viability against PC12 cells injury (Sheng et al., 2017). *Radix Ophiopogonis* is the one of most widely used Chinese herb in Traditional Chinese Medicine (Wang et al., 2015), this polysaccharide protected PC12 cells through suppressing the increase of the intracellular oxidative stress and endoplasmic reticulum stress (Liu and Li, 2018).

In neuronal cells, mitochondria can be found to enhance at areas of increased energy demand, to alter their motility in axons and at synapses in order to maintain energy homeostasis that is essential for synaptic functions (Sheng, 2017). Mitochondria-derived ATP production provides most of the axonal energy, and damaged mitochondria fail to produce ATP. Biological energy deficits and chronic oxidative stress trigger axonal pathology and synaptic dysfunction, thus contributing to pathogenesis of NDs (Theocharopoulou, 2020). Research showed that sulfated chitosan suppressed rotenone – induced – mitochondrial dysfunction, nuclear condensation, and DNA fragmentation in human neuroblastoma SH-SY5Y cell line (Manigandan et al., 2019). Likewise, in rotenone – induced PD animal model, fucoidan extracted from *Laminaria japonica* enhanced mitochondrial respiratory function through the PGC-1α/NRF2 pathway, alleviated DA degeneration and motor impairments (Zhang L. et al., 2018).

Neuroinflammatory responses are also major pathogenic factors in neurodegeneration, and microglia is key participant in neuroinflammatory. Microglia activation and increased inflammatory cytokines have been implicated in the cognitive decline associated with NDs. Microglia activation produces a variety of pro-inflammatory mediators and pro-inflammatory cytokines, plays a crucial role in various NDs (Luo et al., 2013). These cytokines enter the brain, stimulate microglia to trigger neuroinflammatory responses, induce neuronal degeneration and death. Traditional Chinese medicine – *Schisandra chinensis* protected against DA neurodegeneration by suppressing neuroinflammation *via* the BDNF/Nrf2/NF-κB pathway in 6-OHDA-induced PD mice (Yan et al., 2021). And *G. lucidum* prevented the production of microglia-derived proinflammatory and cytotoxic factors in MPP^+^-treated MES 23.5 cell membranes (Zhang et al., 2011).

Apoptosis occurs normally during development and aging and as a homeostatic mechanism to maintain cell populations, including normal cell turnover, proper development and functioning of the immune system, hormone-dependent atrophy, embryonic development and chemical-induced cell death (Elmore, 2007), and apoptosis and caspase-mediated cell death are important mediators of neuronal death in NDs. Traditional herbal medicine has been used for centuries in China to treat PD and remains in use, suggesting that medicinal herbs may be a good source of drug candidates for the treatment of PD (Chen et al., 2007). *Gynostemma pentaphyllum* Makino is a well-known edible and medicinal plant in Asia (Su et al., 2021), its extract polysaccharides decreased Bax/Bcl2 ratio, attenuated the activation of caspase-3/9, and improved PC12 cell viability of MPP^+^ – induced PD cell model (Deng and Yang, 2014). *Lycium barbarum* polysaccharides reduced apoptosis to reverse the decreased viability of PC12 cells in a vitro PD model (Gao et al., 2014).

Autophagy is a cellular basic metabolic process that degrades the aggregated or misfolded proteins and abnormal organelles in cells. The abnormal regulation of neuronal autophagy is accompanied by the accumulation and deposition of irregular proteins, leading to changes in neuron homeostasis and neurodegeneration. Autophagy is a unique mechanism for cells to protect themselves. When autophagy is weakened, abnormal protein aggregation will accelerate the onset of PD; and when autophagy is too strong, it will lead to the autophagy death of nerve cells. Studies show that PTEN-induced putative kinase 1 (PINK1) and E3 ubiquitin protein ligase (Parkin) participate in mitochondrial autophagy, mutations in PINK1 and Parkin genes result in defective mitochondria and trigger early onset PD (Meka et al., 2015). Ren et al. has reported that *G. lucidum* extract attenuated autophagy, and declined in the expression of PINK1 and Parkin, improved behavioral performance in PD model (Ren et al., 2019).

Polymannuronic acid (PM) is a water-soluble homopolysaccharide and can be easily separated *via* pH fractionation from alginate hydrolyzate (Li et al., 2013), it increased gut microbial diversity and fecal SCFAs production, improved the integrity of the intestinal barrier and BBB, prevented DA neuronal loss and improved motor functions in MPTP-induced PD mice (Dong et al., 2020).

To date, a number of literatures have been reported for the possible mechanism of traditional Chinese medicine in treating PD (Rai et al., 2021). As shown in Table 2, current research in PD show that polysaccharides play neuroprotective roles *via* inhibiting cell apoptosis, neuroinflammatory responses and oxidative stress, reversing mitochondrial dysfunction, regulating microbiome and its metabolic product, improving autophagy and other mechanisms.

**TABLE 2:** Polysaccharides and their effects on PD.

Experimental models	Source of polysaccharides	Effects	References
MPTP-induced mouse	*Momordica charantia*	Inhibit oxidative stress and increase DA levels	Guo et al., 2021
MPTP-induced mouse	*spirulina platensis*	Inhibit oxidative stress	Zhang et al., 2015
6-OHDA-induced mouse	*Antrodia camphorata*	Inhibit oxidative stress	Han et al., 2020
H_2_O_2_-induced PC12 cells	*Cynomorium songaricum*	Reduce oxidative stress	Wang F. et al., 2016
H_2_O_2_-induced PC12 cells	*Platycodon grandiflorum*	Attenuate intracellular ROS formation	Sheng et al., 2017
MPP^+^ -induced PC-12 cells	Radix Ophiopogonis	Reverse oxidative stress and ER stress rise	Liu and Li, 2018
Rotenone-induced SH-SY5Ycells	Low molecular weight Sulfated chitosan	Suppress mitochondrial dysfunction	Manigandan et al., 2019
Rotenone-induced Sprague-Dawley Rat	Focoidan	Enhance mitochondrial respiratory function	Zhang L. et al., 2018
6-OHDA-induced mice	*Schisandra chinensis*	Suppress neuroinflammation	Yan et al., 2021
MPP^+^-treated MES 23.5 cell membranes	*Ganoderma lucidum*	prevent the production of microglia-derived proinflammatory	Zhang et al., 2011
MPP^+^-induced PC12 cells	*Gynostemma pentaphyllum*	Inhibit apoptosis	Deng and Yang, 2014
6-OHDA-induced PC12 cells	*Lycium barbarum*	Decrease apoptosis	Gao et al., 2014
MPTP-induced mouse; MPP^+^-induced neuro-2a.	*Ganoderma lucidum*	Attenuate autophagy	Ren et al., 2019
6-OHDA-induced PC12 cells	*Astragalus*	Increase autophagy	Tan et al., 2020
MPTP-induced mouse	Polymannuronic acide	Modulate brain-gut-microbiota axis, increase gut microbial diversity and increase fecal SCFAs production.	Dong et al., 2020

### The Role of Polysaccharides in Alzheimer’s Disease

Alzheimer’s disease is a common degenerative disease of the nervous system with insidious onset and progressive development worldwide. The AD progression develops from initial short-term memory loss to behavioral problems, gradual loss of physical function, and finally death (Tarawneh and Holtzman, 2012). It is believed that the pathogenesis of AD includes the excessive aggregation of Aβ and neurofibrillary tangles formed by hyperphosphorylation of Tau protein (Zhang et al., 2021), release of pro-inflammatory cytokines (Bellucci et al., 2004), mitochondrial dysfunction (Kukreja et al., 2014), oxidative stress (Guix et al., 2012; Wahlster et al., 2013), apoptosis (Zhu et al., 2006), autophagy (Plaza-Zabala et al., 2017), and so on.

The cause of AD remains unclear, and all current medications only relieve the symptoms or delay its development. Aβ is produced and released from the axon terminals of neurons already afflicted with tau pathology (Braak and Del Tredici, 2013). FDA approved AD drugs such as acetylcholinesterase (AChE) inhibitors and *N*-methyl-D-aspartate receptor antagonists only alleviate symptoms in about half of the patients for approximately 6–12 months (Winslow et al., 2011).

In amyloid precursor protein/presenin 1 (APP/PS1), a double transgenic mouse model of AD, *Codonopsis pilosula* polysaccharide decreased the expression of Aβ42 and Aβ40 in the hippocampus and alleviated cognitive impairment through the restoration of synaptic plasticity (Wan et al., 2020). *Maitake* polysaccharide, an edible/medicinal mushroom, activated microglia and astrocytes, promoted the recruitment of microglia to Aβ plaques, enhanced Aβ phagocytosis to reduce Aβ load, increased the number of surviving neurons and maintained the histomorphology of the hippocampus, finally improved learning and memory impairment in APP/PS1 mice (Bai et al., 2019). Furthermore, polysaccharides also have been found to have the same effect in cellular models. *Lycium barbarum* polysaccharide reduced the expression of Aβ42/Aβ40 against β-amyloid peptide neurotoxicity in N2a/APP695 cells (mouse neuroblastoma N2a cells stably expressing human APP695; Wu et al., 2021a). A novel pectin polysaccharide from *Polygala tenuifolia* enhanced the expression of insulin-degradation enzyme and Neprilysin (NEP) to attenuate Aβ42 production and inhibit Aβ42 aggregation (Zeng et al., 2020). Moreover, polysaccharide obtained from *Coptis chinensis* Franch could inhibit the deposition of Aβ *via* the reduction of Aβ-induced toxicity and delaying of the aging in a transgenic *Caenorhabditis elegans* (*C. elegans*) model of AD (Li Y. et al., 2018).

The presence of tau pathology and its correlation with cognitive deficits encouraged the statement of the “tau hypothesis” of AD (Kametani and Hasegawa, 2018). Tau is a microtubule-related protein that aggregates tubulin into microtubules, maintains complex neuronal cell microstructure promoting neuron maturation and regulating synaptic function (Dawson et al., 2001; Castellani and Perry, 2019), changes in synaptic distribution and interruption of interaction with synaptic proteins, damage neuron function and even lead to AD. Previous studies showed that polysaccharides from various plants alleviate cognitive impairment and pathological alterations in AD animal models (Ho et al., 2010; Zhang J. et al., 2016). In an adeno-associated virus serotype 2 (AAV2)-induced expression of human full-length Tau (hTau) in C57/BL6 mice to mimic AD tau pathology, *C. pilosula* polysaccharide significantly increased protein phosphatase-2A (PP2A) activity to attenuate tau phosphorylation in the hippocampus of mice (Zhang Q. et al., 2018). Polysaccharides, extracted from an edible fungus *Pleurotus ostreatus*, decreased Aβ accumulation and tau phosphorylation by increasing the expression of PP2A and glycogen synthase kinase 3beta (GSK3), alleviated cognitive impairment in AD rats (Zhang Y. et al., 2016). An intranasally applied polysaccharide Mini-GAGR can cross BBB to increase antioxidant enzymes in the hippocampus and cortex, decrease phosphorylated-tau (p-tau) and improve memory in AD mice (Murphy et al., 2018). Similarly, another 4.7 KD polysaccharide Midi-GAGR also penetrated the BBB to reduce free reactive radicals and microglia cells, increased neurite outgrowth and phosphorylated cAMP responsive element binding protein, decreased hyperphosphorylated tau for neuroprotection in the transgenic AD mouse model (Makani et al., 2016).

Acetylcholine (Ach) is a signal transmitter of cholinergic neurons, its metabolic processes influence learning and memory in AD brain (Ferreira-Vieira et al., 2016). A commonly used traditional Chinese herb *Angelica sinensis* is widely used for nourishing the blood. *Angelica* polysaccharide regulated the balance of neurotransmitters by decreasing AChE level and increasing Ach and acetyltransferase (ChAT), eventually meliorated spatial learning and memory deficiency in AD rats (Du et al., 2020). Porphyran, from red algae *Pyropia haitanensis*, increased ChAT activity and decreased AChE activity in the cortical and hippocampal, significantly ameliorated the learning and memory impairment induced by Aβ1-40 of AD mice (Zhang et al., 2020c). The changes of chAT and AchE activity are considered to be important indicators to indirectly reflect the cholinergic biochemical changes in AD (Mantzavinos and Alexiou, 2017).

In Aβ-induced AD rats, marine brown seaweed fucoidan, a complex sulfated polysaccharide, increased of Bcl2/Bax ratio and decreased of caspase-3, inhibited cell apoptosis followed by improving the learning and memory abilities (Gao et al., 2012). Non-saponin fraction with rich polysaccharides from ginseng ameliorated mitochondrial deficit in Aβ-treated HT22 cells and enhanced and restored the cognitive function of healthy and AD mice (Shin et al., 2021). Furthermore, study has shown that many natural polysaccharides are also called immune polysaccharides due to their immunological activity. Polysaccharides from *Schisandra Chinensis Fructus* significantly reduced the deposition of Aβ, improved the cognition and histopathological changes *via* downregulating the expression of pro-inflammatory cytokines such as IL-1β, IL-6, and TNF-α, and the activation of glial cells in the hippocampus of AD mice (Xu et al., 2019). Moreover, *G. lucidum* polysaccharides *via* oral administration promoted neural progenitor cell proliferation to enhance neurogenesis and alleviated cognitive deficits in transgenic AD mice, and also improved locomotor functions and prolonged the life span of *Drosophila* Aβ42-AD model (Huang et al., 2017).

As reported, the promotion of autophagy initiation and autolysosome formation can reduce the aggregate-prone proteins aggregation and its neurotoxicity in AD model (Nixon, 2013). Two trehalose analogs including lactulose and melibiose increase autophagy by upregulating the LC3II/LC3I and downregulating the p62, decrease neuroinflammation, eventually attenuated the short-term memory and the learning retrieval in AD mice (Lee et al., 2021). Moreover, In AD-like symptom C57BL/6J mice, polysaccharides from *Taxus chinensis* var. *mairei* Cheng et L.K.Fu (Taxaceae) inhibited oxidative stress, restored the impaired learning and cognitive function *via* regulating the expression of NF-E2-related factor 2 (Nrf2) which plays an important role in cell defense against oxidative stress in CNS (Zhang et al., 2020a). A fungal species *Inonotus obliquus* polysaccharide also decreased oxidative stress *via* Nrf2 signaling, improved the pathological behaviors related memory, and cognition in APP/PS1 mice (Han et al., 2019).

In conclusion, AD seriously threatens aged people’s health. As shown in Table 3, we speculate that polysaccharides can reduce oxidative stress, apoptosis and neuroinflammation, regulate the balance of neurotransmitters, increase autophagy, ultimately decrease Aβ peptide formation and tau phosphorylation, alleviate cognitive impairment in AD models. Consequently, it is plausible to recommend polysaccharide as one of the promising tools in the development of drug therapy for AD.

**TABLE 3:** Polysaccharides and their effects on AD.

Experimental models	Source of polysaccharides	Mechanism	References
APP/PSI transgenic mice	*Codonopsis pilosula*	Restore synaptic plasticity	Wan et al., 2020
APP/PSI transgenic mice	*Maitake*	Promote the recruitment of microglia to Aβ plaques	Bai et al., 2019
N2a/APP695 cells	*Lycium barbarum*	Reduce Aβ peptide neurotoxicity	Wu et al., 2021a
CHO/APPBACE1 and HEK293-APPsw cells	*Polygala tenuifolia*	Enhance main enzymes (IDE and NEP) involved in Aβ degradation expression	Zeng et al., 2020
transgenic *C. elegans*	*Coptis chinensis*	Reduce Aβ-induced toxicity	Li Y. et al., 2018
AAV2-hTau-infected C57/BL6 mice	Codonopsis pilosula	Attenuate Tau phosphorylation by increasing PP2A activity	Zhang Q. et al., 2018
Male Wistar rats	*Pleurotus ostreatus*	Decrease Tau phosphorylation by elevating PP2A and reducing APP, BACE1, GSK3 expression	Zhang Y. et al., 2016
3xTg-AD mice	Mini-GAGR	Increase antioxidant enzymes decrease and p-Tau	Murphy et al., 2018
3xTg-AD mice	Midi-GAGR	Penetrate the BBB to reduce free reactive radicals and microglia cells	Makani et al., 2016
Male Sprague-Dawley rats	*Angelica sinensis*	Regulate the balance of neurotransmitters	Du et al., 2020
Aβ1-40 of AD mice	Porphyran	Increase ChAT activity and decreases AChE activity	Zhang et al., 2020c
Sprague-Dawley rats	Fucoidan	Inhibit cell apoptosis	Gao et al., 2012
Male KM mice	*Schisandra Chinensis Fructus*	Decrease neuroinflammation	Xu et al., 2019
transgenic AD mice; transgenic Drosophila	*Ganoderma lucidum*	Enhance neurogenesis	Huang et al., 2017
C57BL/6J mice	lactulose and melibiose	Attenuate autophagy	Lee et al., 2021
C57BL/6J mice	Taxus chinensis var. mairei Cheng et L.K.Fu	Decrease oxidative stress	Zhang et al., 2020a
APP/PSI transgenic mice	*Inonotus obliquus*	Inhibit oxidative stress	Han et al., 2019

### Huntington’s Disease

Huntington’s disease is a single-gene autosomal dominant neurodegenerative disorder with the disease-causing gene IT-15 (the *HTT* gene). The HD gene has been mapped in 4p16.3, which is abnormally amplified in CAG trinucleotide repeats in HD. When the number of CAG copies is abnormally increased due to gene mutation, the polyglutamine (polyQ) chain at the amino-terminal of the mutant Htt (mHtt) protein is prolonged and abnormally folded. The β lamellae structure is formed to cause the loss of normal function and toxic effect of mHtt protein (Southwell and Patterson, 2011). HD usually presents in midlife with chorea, dementia, and weight loss, with death typically occurring 15–20 years after symptom onset (Walker, 2007), the dance-like movements that are a primary clinical symptom of HD. Nonetheless, the therapies currently available to HD patients only moderate symptom relief and do not affect disease progression.

A prominent pathological feature of HD is the accumulation of mHtt in neurons, and mHtt plays a cytotoxic role by affecting transcription, mitochondrial function, synaptic transmission, and axon transport (Ramakrishnan and Gupta, 2021). Compared with ordinary Htt, mHtt has a longer polyQ (>36) extension at the N-terminal (Ross, 2002). mHtt undergoes proteolysis, misfolding, accumulation, self-aggregation, and eventually forms inclusion bodies (Cortes and La Spada, 2014). mHtt engages in various aberrant interactions that lead to the pathological gain of toxic functions and loss of normal functions. The study found that reduced AKT phosphorylation and inhibited AKT activity are involved in mHtt-induced cell death. *Lycium barbarum* polysaccharide alleviated the cytotoxicity of mHtt by activating AKT and reduceing mHtt levels in HD-transgenic mice (Fang et al., 2016). *Astragalus membranaceus* polysaccharide reduced polyQ aggregation and alleviated the associated neurotoxicity in *C. elegans* (Zhang et al., 2012). *Peganum harmala* L. polysaccharide inhibited polyQ aggregation through proteasome-mediated protein degradation, alleviated the neurotoxicity of *C. elegans* (Guo et al., 2020).

Soluble and aggregative mHtt has cytotoxicity, manifests primarily as excessive intracellular oxidative stress response leading to cell apoptosis in the mitochondrial pathway (Reijonen et al., 2008; Ayala-Peña, 2013), increases expression of apoptosis-related genes (Bae et al., 2005), and continuous degeneration of neurons caused by cell cycle re-entry (Pelegrí et al., 2008). Polysaccharide is capable of inhibiting behavioral dysfunction mediated by reducing polyQ aggregation. The study found that a kind of traditional Chinese medicine – epimedium is a genus of plants in the family *Berberidaceae*, and its polysaccharide enhanced antioxidant enzyme activities, decreased lipid peroxidation product and oxidative stress, increased the survival rates, attenuated behavioral dysfunction in polyQ transgenic *C. elegans* HD models (Xiang et al., 2017). In the similar HD models, the polysaccharide from a medicinal mushroom *Dictyophora indusiata* was capable of reducing ROS levels and alleviating chemosensory behavior deficits (Zhang J. et al., 2016). 3-nitropropionic acid (3-NP) induces a selective striatal pathology in HD, and has been widely used as an animal model of HD. Ginseng saponins decreased intracellular Ca^2+^ and cytotoxicity of striatal neurons, significantly improved behavioral impairment, and extended the survival of 3-NP-induced HD rats (Kim et al., 2005). Furthermore, polysaccharides and their effects on HD are shown in Table 4.

**TABLE 4:** Polysaccharides and their effects on HD.

Experimental models	Source of polysaccharides	Effects	References
HD-transgenic mice	*Lycium barbarum*	Alleviate the cytotoxicity of mHtt	Fang et al., 2016
*C. elegans*	*Astragalus membranaceus*	Reduce polyQ aggregation and alleviate neurotoxicity	Zhang et al., 2012
*C. elegans*	*Peganum harmala*	Inhibit polyQ aggregation and alleviate the neurotoxicity	Guo et al., 2020
*C. elegans*	*epimedium*	Reduce oxidative stress	Xiang et al., 2017
transgenic *C. elegans*	*Dictyophora indusiata*	Reduce oxidative stress	Zhang J. et al., 2016
3-NP-induced SD rats	Ginseng saponins	Alleviate cytotoxicity of striatal neurons	Kim et al., 2005

## Discussion

Previous studies suggest that polysaccharides are employed to protect against NDs. Herein, this review presents multiform polysaccharides protect against NDs *via* mediating various mechanisms. Despite many signaling pathways involved in, the common mechanisms of polysaccharides mainly include neuroinflammation, oxidative stress, autophagy, apoptosis and mitochondrial dysfunction in PD and AD for neuroprotective effects. And, HD related studies are still rare. In the above literatures, a majority of polysaccharide in NDs have been studied *in vitro*. Most polysaccharides have a poor BBB permeability besides chitosan nanoparticles and LPS. Therefore, it is rarely reported that the polysaccharides directly activate polysaccharides receptors in the CNS of NDs. How do the polysaccharides work in the CNS? Up to now, only polysaccharide receptor TLR-4 has been reported to be bind with LPS to increase accumulation of Aβ in AD. Based on above studies and the metabolism of polysaccharides, the polysaccharides may directly penetrate BBB to act on the polysaccharide receptors or mediate intestinal microorganism and metabolites to activate downstream signaling for neuroprotective effect in NDs. Whereas, the related studies are still limited.

Most NDs patients develop gastrointestinal motility abnormalities and constipation, hence intestinal flora might be closely related to the development of NDs. The imbalance also affects the intestinal mucosal barrier, triggers intestinal and peripheral neuroinflammatory responses. These phenomena may lead to neuroinflammation or neurodegeneration in CNS (Pellegrini et al., 2018). Polysaccharide molecules are closely related to the regulation of intestinal flora, the role of the microbiota provides new therapeutic targets for NDs (Sun et al., 2020). Nerves, immunity, and the endocrine system could alter the microbiota-gut-brain axis. The intestine, the brain and immune system dysfunctions promotes the development and progression of NDs. The intestinal microbiota and its primary metabolites SCFAs affect brain activity and behavior *via* endocrine, vagus nerve, immune pathways and other humoral pathways (Shaik et al., 2020). SCFAs promote the development and growth of brain microglia, play a central role in brain development and the maintenance of CNS homeostasis (Erny et al., 2015). Natural polysaccharides have received increasing attention and have become popular dietary nutrients because of their various biological functions. Some natural polysaccharides are favorable for the proliferation of SCFA-producing bacteria, the presence of which improves the intestinal microenvironment. Moreover, natural polysaccharides also suppress excessive inflammatory responses by improving the intestinal microbiota composition, promoting SCFAs production, strengthening intestinal barrier function and reducing pro-inflammatory mediators (Tang et al., 2019). A plant polysaccharide from *Cistanche deserticola* improves cognitive function in mice in a D-galactose induced aging model by restoring homeostasis of the gut-microbiota-brain axis (Gao et al., 2021). Therefore, polysaccharides may mediate intestinal flora and related metabolites against NDs.

The natural polysaccharides contain polysaccharides and their derivatives (such as nanoparticles). Using polysaccharides nanoparticles for drug delivery can enhance the aqueous solubility of the drug (Kang et al., 2015). Additionally, specific polysaccharides can provide targeting mechanisms due to receptor recognition and binding (Yadav et al., 2008), mucosal adhesion and transport (Feng et al., 2015), site specific enzymatic degradation (Castelli et al., 2008). Polysaccharide-based drug delivery, specially, polysaccharide nanoparticle has been improved the uptake and specificity of drugs and the bioavailability of poorly soluble drugs. Such as, polysaccharide chitosan as the only essentially natural cationic polysaccharide is useful for chemical modification and electrostatic interactions in drug delivery systems in NDs (Sarvaiya and Agrawal, 2015; Liu et al., 2017; Raj et al., 2018). Starch is a glucose-based polysaccharide produced by many green plants to store energy, often as an excipient in pharmaceutical products, have been used to modify the properties of drug delivery systems to improve sustained release and directly to enhance drug delivery in PD (Odeniyi et al., 2018; Becker et al., 2021). An energy storage polysaccharide Inulin is found in plants of the *Compositae* family, and also indigestible to humans and digested by the enzymes of bacteria in the gut, allowing to be exploited for colon targeted delivery (Castelli et al., 2008). Polysaccharides represent a platform for therapeutic delivery applications, and these multiple functions are likely to be increasingly applied in the future.

There are growing evidences on the fact that distinct immune responses, involving the adaptive as well as the innate immune system, are crucially implicated in NDs (Chitnis and Weiner, 2017), and immunotherapy is one of the most studied therapeutic strategies in NDs (Ciccocioppo et al., 2020). Moreover, microglia and astrocytes protect the brain from infectious agents, while their prolonged activation causes neuroinflammation that promote neurodegeneration (Barbalace et al., 2019). Neuroinflammation significantly initiates and enhances neurodegenerative pathological change, natural anti-inflammatory compounds may be good candidates for the development of successful therapeutic strategies. Polysaccharides have significant pharmaceutical importance due to their strong anti-inflammatory and immunomodulatory properties (Hou C. et al., 2020). The polysaccharides in AD, PD, and HD share a common mechanism – oxidative stress. Growing reports have demonstrated that oxidative stress is implicated in the development and progression of many chronic diseases, and oxidative stress is a condition of imbalance between ROS formation and cellular antioxidant capacity due to enhance ROS generation or dysfunction of the antioxidant system. Many polysaccharides are reported to have potent reducing power and free radical scavenging ability *in vitro*, and reduce the levels of ROS and associated peroxidation products in cellular and animal models under oxidative stress. Furthermore, oxidative stress can interact with many other stresses to induce neurodegeneration, so antioxidant polysaccharides play significant role as a pharmacological potential in treatment NDs.

In addition, the polysaccharides also develop for clinical applications. Acemannan, the main bioactive polysaccharide of *Aloe vera*, is a neuroprotective immunomodulator and antioxidant, and improved the cognitive performances of middle-aged patients suffering from mental fatigue (Liu et al., 2019). Furthermore, chitosan as a biomaterial for the development of a flexible, thin film, laser-activated surgical adhesive termed surgical adhesive, are FDA approved and successfully used in a variety of biomedical applications and products. Therefore, chitosan is an alternative to microsurgery for peripheral nerve reconstruction (Foster and Karsten, 2012). Chitosan nerve tube can improve peripheral sensory nerve regeneration of the patients in traumatic sensory nerve lesions of the hand (Neubrech et al., 2018). So polysaccharides and their mechanisms deserve further study as potential candidates for preventing and treating NDs.

## Author Contributions

YW wrote the manuscript. NZ and JY contributed to writing and review by the manuscript. RC, QW, and XC were implied in collecting and sorting literatures and references. ZY wrote a small part and offered some ideas for the first manuscript, and improved language, revised, refined the manuscript in the process of revision. All authors read and approved the final manuscript.

## Conflict of Interest

The authors declare that the research was conducted in the absence of any commercial or financial relationships that could be construed as a potential conflict of interest.

## Publisher’s Note

All claims expressed in this article are solely those of the authors and do not necessarily represent those of their affiliated organizations, or those of the publisher, the editors and the reviewers. Any product that may be evaluated in this article, or claim that may be made by its manufacturer, is not guaranteed or endorsed by the publisher.
